# Muscle Mass in Children and Adolescents: Proposed Equations and Reference Values for Assessment

**DOI:** 10.3389/fendo.2019.00583

**Published:** 2019-08-28

**Authors:** Marco A. Cossio Bolaños, Cynthia Lee Andruske, Miguel de Arruda, Jose Sulla-Torres, Camilo Urra-Albornoz, Margot Rivera-Portugal, Cristian Luarte-Rocha, Jaime Pacheco-Carrillo, Rossana Gómez-Campos

**Affiliations:** ^1^Programa de Doctorado en Ciencias de la Actividad Física, Universidad Católica del Maule, Talca, Chile; ^2^Centro de Investigación CINEMAROS, Arequipa, Peru; ^3^Faculty of Physical Education, State University of Campinas, Campinas, Brazil; ^4^Universidad Católica de Santa María, Arequipa, Peru; ^5^Escuela de Kinesiología, Facultad de Salud, Universidad Santo Tomás, Talca, Chile; ^6^Universidad Nacional de San Agustín de Arequipa, Arequipa, Peru; ^7^Universidad San Sebastian, Concepción, Chile; ^8^Departamento de Ciencias de la Educación, Universidad de Bio Bio, Chillán, Chile; ^9^Departamento de Diversidad e Inclusividad Educativa, Universidad Católica del Maule, Talca, Chile

**Keywords:** lean mass, references, children, adolescents, DXA

## Abstract

**Objectives:** The goal of this study was to develop regression equations to estimate LM with anthropometric variables and to propose percentiles for evaluating by age and sex.

**Methods:** A cross sectional study was conducted with 2,182 Chilean students (1,347 males and 835 females). Ages ranged from 5.0 to 17.9 years old. A total body scan was carried out with the double energy X-ray anthropometry (DXA) to examine and measure lean muscle mass of the entire body. Weight, height, and the circumference of the relaxed right arm were also measured.

**Results:** Four anthropometric equations were generated to predict lean mass for both sexes (*R*^2^ = 83–88%, SEE = 3.7–5.0%, precision = 0.90–0.93, and accuracy = 0.99). The Lambda-mu-sigma method was used to obtain the sex-specific and age-specific percentile curves of lean mass (p3, p5, p10, p15, p25, p50, p75, p85, p90, p95, and p97).

**Conclusion:** The four proposed equations were acceptable in terms of precision and accuracy to estimate lean mass in children and adolescents. The percentiles were created by means of anthropometric equations and real values for DXA. These are fundamental tools for monitoring LM in Chilean children and adolescents of both sexes.

## Introduction

Muscle mass or lean mass (LM) is an essential component of body composition. Furthermore, it plays an important role in maintaining posture and normal movement for adults as well as for children and adolescents (1).

Actually, measurement of the LM is considered to be an important component of the nutritional status of children and adolescents. Increasingly, it is recognized as an independent marker of metabolic health (2) and physical performance that allows verification of changes in the LM due to the effects of physical training (3).

In general, various methods exist to evaluate the LM: criterion and/or gold standard. For example, some of these include the multiple magnetic resonance imaging scan (MRI), computerized axial tomography scan (CAT) (4), total body potassium analysis (5), bio-electric impedance (6), and the Dual X-ray Absorptiometry scan (DXA) (7), among others.

The DXA is the preferred technological method for detecting body composition that provides information about a body model with three components: lean mass (LM), fat mass (FM), and bone mass (BM). This method stands out as one of the most accurate and applicable for assessing bone mineralization and body composition in pediatric populations (8).

In fact, despite DXA's utility and significant advantages, it has some limitations above all when it is necessary to carry out population studies, especially in primary care. This is due, particularly, to the high cost of the exam, the necessity for trained certified professional operators, and the need for available specialized infrastructure that limit its use to laboratory conditions (9, 10).

In this context, estimations of LM based on anthropometric variables are potentially useful, especially when studying young individuals during the growth phase and biological maturation (10). These indicators together may specifically be adjusted to each body size to more accurately identify the LM. Moreover, it may be a low cost and easy to use alternate method in epidemiological contexts (11).

As a result, the weight and height variables are continually used as indicators of physical growth and nutritional status in the short term and long run. In addition, arm circumference is used as an alternative means of detecting malnutrition since it provides estimates for muscle mass and fat reserves (12).

Thus, the relationship of these variables together could help develop equations in order to estimate the muscle mass of children and adolescents in large populations, reducing evaluation costs and time. Essentially, this would avoid exposure to radiation in the future.

As a result, LM is important in studying nutritional, physiological, and metabolic processes (13). In addition, LM plays a fundamental role in growth maintenance, normal development, and systemic glucose metabolism in children (14). Based on the need for a non-invasive method to estimate LM in children and adolescents in Chile, this study had two objectives: (a) develop regression equations to estimate LM using anthropometric variables and (b) propose percentiles for evaluating LM based on age and sex.

Furthermore, although a number of international studies have been carried out in recent years with some of these characteristics, to date, no studies have been conducted in Chile using LM, anthropometric variables, regression equations, and percentiles (2, 14, 15). It is important to highlight that the reference data may provide relevant information, not only for DXA as a laboratory method, but also for using regression equations based on anthropometric variables as a field method.

In general, independent from the uses and applications, both objectives may be useful in the health sciences and sports sciences contexts. In addition, the results may facilitate calculations for professionals working in medical centers, schools, laboratories, nutrition, and other types of institutions.

## Materials and Methods

### Population

For this research, a descriptive cross-sectional study was carried out. The universal sample population was composed of 21,120 students. The sample was selected probabilistically (random) CI:95%. The size of the estimated sample was 10.4% resulting in 1,347 males (6.4%) and 835 females (4.0%). Ages ranged from 5.0 to 18.9 years. The students recruited for this research attended 12 public schools in the Maule Region of Chile. This region is located in the central valley of Chile. The primary industry is agriculture with a Human Development Index for 2012 of 0.72 for the Maule Region.

Permission to conduct the study and collect information was requested from the Direction of the Administration of Municipal Education (Municipal Administration of Education) of Talca (DAEM-Chile) and the administration of each school. Children and adolescents included in the study were those whose parents and/or guardians agreed by signing an informed consent form as well as those meeting the established age requirements for the study. Students who smoked or those with one or more bone fractures occurring during the last 3 months were excluded from the study.

This research project was approved by the Ethics Committee of the Universidad Autónoma de Chile (protocol no. 238/2013). The experimental procedure was based on the Helsinki Declaration Accord (World Medical Association) for human subjects.

### Procedures

The decimal age of each student was recorded (birth date and evaluation date). All students were put into 14 categories by age and in 1 year intervals (for example, 5.0 to 5.9 years, 6.0 to 6.9 years).

Evaluation of the anthropometric variables and the dual energy X-ray absorptiometry (DXA) scan were carried out in a closed laboratory with a constant temperature between 20 and 24°C. All measurements were taken during the morning and the afternoon (8:30 a.m. to 12 noon and 14:00 to 18:00 h) from Monday to Friday during the months of March to November 2015.

The standard protocol proposed by the “International Cineanthropometry Working Group” and as described by Ross and Marfell-Jones (16) was used to evaluate the anthropometric variables. Anthropometric variables were measured when the students were barefoot and with the least amount of clothing possible (shorts and T-shirt). Students were weighed using a Tania (United Kingdom Ltd.) digital scale with an accuracy of 1.0 kg. Standing height was measured according to the Frankfurt Plane with a portable stadiomete (Hamburg Seca, Ltd.) with a 0.1 mm. accuracy. Sitting height (cephalic trunk height) was evaluated while the subject was sitting on a wooden bench with a height of 50 cm. The measurement scale consisted of 0 to 100 cm with an accuracy of 1 mm. The circumference of the relaxed right arm was taken with a tape measure (cm) while the subject maintained a relaxed supine position with arms hanging by the sides of the body. The measurement was taken midway between the tip of the acromion and olecranon process. Somatic maturity was predicted by using a regression equation proposed by Mirwald et al (17). For this calculation, age, weight, standing height, and sitting height were used. Body Mass Index (BMI) was obtained from the weight and height using the formula proposed by Quetelet: [BMI = weight (kg)/height (m)^2^]. All of the anthropometric variables were measured two times. The technical error of measurement (|TEM) for weight, standing height, sitting height, and circumference of the right arm oscillated between 1.0 and 2.5%.

A total body scan was carried out using double energy X-ray absorptiometry DXA (Lunar Prodigy; General Electric, Fairfield, CT). Lean mass, fat mass, bone mass, and % of body fat of the total body values were recorded. For this procedure, the subjects needed to lie down on an examination platform in a supine position with the arms and legs extended. The ankles were tied together with a Velcro belt to ensure that a standard position was maintained. The subjects were warned about not using jewelry and the presence of any type of metal in or on the body that might impede the scan.

The evaluation of the anthropometric variables and the DXA total body scan lasted ~10–12 min for each student. Ten percent of the student sample studied (135 subjects) was scanned two times to guarantee the technical error of measurement (TEM). DXA was performed by a technician with significant experience. The equipment was calibrated daily according to the manufacturer's instructions.

### Statistics Analysis

The normality of the data was verified by the Kolmogorov-Smirnov (K-S) test. Descriptive statistical analysis of the arithmetic average, standard deviation, and range were carried out. The *t*-test for independent samples to determine the differences between both sexes was carried out to verify the differences between the values of the predictor equations and the DXA reference. The relationship between the variables was verified by using Pearson's correlation coefficient. Four multiple regression equation models were developed to predict the lean mass (2 for males and 2 for females). The models were generated with the total sample studied (1,347 males and 845 females). The multiple regression analysis process was carried out in steps until the best combination of predictor variables was identified for lean mass by sex. The equations were analyzed using % of explanation of *R*^2^ and the Standard Error of Estimation (SEE). In addition, multi-co-linearity was analyzed using the variance inflation factor (VIF) and tolerance. Calculations were carried out with SPSS 18.0. In all cases, *p* < 0.001 was adopted. The concordance correlation coefficient (CCC) was calculated using Lin's (18) approach. Calculations were carried out using MedCalc Statistical Software v.11.1.0, 2009 (Mariakerke, Bélgium). Precision (p) and accuracy (A) were determined with the estimated values of LM and DXA criterion. The LMS (19) statistical method was used to create reference curves based on the regression equations generated and by the DXA criterion method based on age and sex. The LMS technique was used to estimate three parameters: median (M), coefficient of variation (S), and the Box-Cox power of transformation (L). These three parameters varied according to age.

## Results

The characteristics of the anthropometric variables and the body composition of both sexes are illustrated in Table 1. No significant differences occurred in chronological age and BMI (*p* > 0.05). However, significant differences emerged between APHV, weight, standing height, sitting height, and arm circumference (*p* < 0.05). The relationship between the muscle mass and the anthropometric variables are shown in Table 2. In all cases, the correlations were significant (*p* < 0.001), and they varied between *r* = 0.67 and 0.91.

**Table 1:** Anthropometric and body composition characteristics of both sexes.

**Variables**	**Males**		**Females**		**Total (*****n*** **=** **2,182)**	
	**X**	**SD**	**X**	**SD**	**X**	**SD**
Chronological age (Years)	13.3	3.7	12.3	3.7	12.9	3.7
Maturity offset, (APHV)	14.9	0.8	11.4	0.7	13.9	1.8
**ANTHROPOMETRY**						
Weight (kg)	53.9	19.2	48.3	16.5^*^	51.7	18.4
Standing height (cm)	155.2	19.7	147	15.4^*^	152.1	18.6
Sitting height (cm)	81.1	10.4	77.3	8.4^*^	79.7	9.9
BMI (kg/m^2^)	21.6	4.3	21.6	4.5	21.6	4.4
AC (cm)	24.8	4.4	24.4	4.1^*^	24.7	4.3
**BODY COMPOSITION (DXA)**						
Bone Mass (Kg)	2.1	0.8	1.7	0.5^*^	1.9	0.7
Fat mass (kg)	13.3	7.4	16.9	8.0^*^	14.7	7.8
Lean Mass (kg)	38.7	14.4	29.8	9.1^*^	35.3	13.3
Fat percentage (% G)	24.8	8.8	34	6.3^*^	28.3	9.1

*AC, Arm circumference; SD, Standard deviation; DXA, double energy X-ray absorptiometry*,

* *p <0.05*.

**Table 2:** Correlation between anthropometric variables with lean mass in children and adolescent (AC, Arm circumference).

**Variables**	**Age (years)**	**Weight (kg)**	**Height (cm)**	**AC (cm)**	**Lean mass (kg)**
Age (years)	x	(0.750^**^)	(0.827^**^)	(0.668^**^)	(0.765^**^)
Weight (kg)	0.891^**^	x	(0.831^**^)	(0.874^**^)	(0.886^**^)
Height (cm)	0.920^**^	0.863^**^	x	(0.96^**^)	(0.856^**^)
AC (cm)	0.715^**^	0.866^**^	0.718^**^	x	(0.770^**^)
Lean Mass (kg)	0.880^**^	0.892^**^	0.906^**^	0.762^**^	x

Values in parentheses (females); AC, Arm circumference;

** *p = 0.001*.

Four regression equations were developed to estimate the muscle mass of children and adolescents of both sexes (Table 3). The tolerance values for all of the equations varied from 0.12 to 0.29 and the VIF between 3.08 and 8.21. Co-linearity did not occur in any of the models (equations) generated. In general, the four equations created showed an explanatory power of 76–84%. In addition, the equations were significant: (*p* < 0.001). Males demonstrated a SEE of <5% while the SEE for the females was <3.7%.

**Table 3:** Regression equations for estimating muscle mass from anthropometric variables.

***N*°**	**Equations**	**T**	**VIF**	**R**	**R^**2**^**	**SEE**	**p**
**MALES (*****n*** **=** **1,347)**							
1	LM = −28.669+0.887^*^age+0.298^*^Weight+0.255^*^Height			0.936	0.876	5.071	0.000
	Age	0.148	6.754				
	Weight	0.246	4.066				
	Height	0.122	8.211				
2	LM = −25.512+1.088^*^age+0.297^*^Weight+0.037^*^AC+0.405^*^Heigh			0.935	0.875	5.08	0.000
	Age	0.185	6.754				
	Weight	0.130	4.066				
	Arm Circunference	0.245	8.211				
	Height	0.165	8.210				
**FEMALES (*****n*** **=** **835)**							
3	LM = −16.264+0.182^*^age+0.302^*^Weight+0.198^*^Height			0.912	0.832	3.71	0.000
	Age	0.303	3.298				
	Weight	0.296	3.375				
	Height	0.214	4.671				
4	LM = −11.504+0.271^*^age+0.393^*^Weight-0.10^*^AC+0.292^*^Height			0.909	0.827	3.78	0.000
	Age	0.324	3.089				
	Weight	0.152	6.565				
	Arm Circunference	0.234	4.265				
	Height	0.254	3.931				

*T, Tolerance; VIF, variance inflation factor; SEE, Standard estimation error; AC, Arm circumference*.

Table 4 illustrates the definition of the degree of agreement, the reference method, and the four proposed equations. No significant differences occurred between the average values of the reference method and equations 1 and 2 in males and equations 3 and 4 for females (*p* < 0.05). The CCC values for equations 1 and 2 for males were from 0.93. For females, in equations 3 and 4, the CCC values were from 0.90. Both groups showed a high degree of agreement. Furthermore, in general, the precision of the equations varied from 0.91 to 0.94, and accuracy was from 0.91.

**Table 4:** Values of the desirable reproducibility index (DRI) for defining agreement between the DXA reference method and the proposed equations.

**Equations**	**X**	**SD**	**Min**	**Max**	**Desirable reproducibility index**		
					**CCC**	**p**	**A**
**MALES**							
DXA Reference LM	38.7	14.4	11.4	71.8	–	–	–
Equation 1	38.7	13.4	6.1	67.5	0.9337 (IC:0.9266 to 0.9401)	0.936	0.998
Equation 2	38.7	13.4	5.3	67.0	0.9334 (IC:0.9263 to 0.9398)	0.936	0.998
**FEMALES**							
DXA Reference LM	29.7	9.1	0.3	55.9	–		
Equation 3	29.7	8.3	7.9	52.2	0.9084 (IC:0.8961 to 0.9193)	0.912	0.996
Equation 4	31.0	9.1	8.7	56.1	0.9008 (IC:0.8874 to 0.9128)	0.909	0.991

*LM, Muscle mass (lean mass); CCC, Correlation coefficient of agreement; P, Precision; A, Accuracy; SD, Standard deviation; Min, Minimum; Max, Maximum; CI, Confidence interval; and DXA, double energy X-ray absorptiometry*.

The values for the percentiles of the LM determined by the DXA and the anthropometric equations are displayed in Tables 5, 6. In all cases, and in both sexes, the median values increased as age advanced. In males (Table 5), small discrepancies emerged between the reference method medians and the regression equations 1 and 2. At ages 5.0 and 6.0 years old, values for the reference were higher: 2.6 to 4.7 kg in relation to both equations. After age 7.0 to 18.9 years old, these discrepancies were reduced to values between −1.8 and 2.5 kg. In females (Table 6), the discrepancies were minimal in all age ranges. For example, in equation 3, the LM discrepancy was between −0.7 and 1.5 kg, and in equation 4, it was between −1.8 and 0.8 kg. On the other hand, significant differences occurred between both sexes commencing at age 11.0 until 18.9 years old. Males showed higher LM, DXA, and regression equation vales (*p* < 0.05) than did the females in this study.

**Table 5:** LMS values and percentile distributions for evaluating muscle mass with DXA and anthropometric equations in males.

						**Males**								
**Age**	**L**	**M**	**S**	**P3**	**P5**	**P10**	**P15**	**P25**	**P50**	**P75**	**P85**	**P90**	**P95**	**P97**
**LEAN MASS BY DXA**														
5.0–5.9	−1.61	15.79	0.18	12.1	12.4	13.0	13.4	14.2	15.8	18.0	19.7	21.0	23.5	25.6
6.0–6.9	−1.24	17.66	0.18	13.3	13.7	14.4	14.9	15.8	17.7	20.2	21.9	23.3	25.7	27.6
7.0–7.9	−0.88	19.47	0.19	14.3	14.8	15.7	16.3	17.3	19.5	22.2	24.0	25.4	27.8	29.6
8.0–8.9	−0.53	21.40	0.19	15.5	16.1	17.0	17.8	18.9	21.4	24.4	26.3	27.7	30.0	31.7
9.0–9.9	−0.17	23.69	0.19	16.7	17.5	18.6	19.5	20.9	23.7	27.0	29.0	30.4	32.7	34.3
10.0–10.9	0.22	26.53	0.19	18.3	19.2	20.6	21.7	23.3	26.5	30.1	32.2	33.7	35.9	37.5
11.0–11.9	0.60	30.11	0.19	20.2	21.4	23.2	24.5	26.4	30.1	34.0	36.2	37.7	40.0	41.5
12.0–12.9	0.98	34.38	0.18	22.6	24.1	26.3	27.9	30.1	34.4	38.6	40.9	42.4	44.7	46.2
13.0–13.9	1.34	39.07	0.17	25.3	27.2	29.9	31.8	34.4	39.1	43.6	45.9	47.5	49.8	51.3
14.0–14.9	1.63	43.74	0.16	28.5	30.7	33.8	35.9	38.7	43.7	48.4	50.8	52.4	54.7	56.1
15.0–15.9	1.86	47.90	0.15	32.0	34.3	37.7	39.8	42.8	47.9	52.6	54.9	56.5	58.7	60.1
16.0–16.9	2.06	51.11	0.14	35.4	37.8	41.1	43.2	46.1	51.1	55.6	57.9	59.4	61.5	62.8
17.0–17.9	2.24	53.50	0.12	38.7	40.9	44.1	46.1	48.8	53.5	57.7	59.8	61.2	63.2	64.4
18.0–18.9	2.42	55.59	0.11	42.0	44.0	46.9	48.8	51.3	55.6	59.5	61.4	62.7	64.5	65.6
**LEAN MASS FOR EQUATION 1**														
5.0–5.9	0.060	11.054	0.213	7.4	7.8	8.4	8.8	9.6	11.1	12.8	13.8	14.5	15.6	16.4
6.0–6.9	0.024	14.548	0.200	10.0	10.5	11.2	11.8	12.7	14.5	16.6	17.9	18.8	20.2	21.2
7.0–7.9	0.025	17.891	0.187	12.6	13.1	14.1	14.7	15.8	17.9	20.3	21.7	22.7	24.3	25.4
8.0–8.9	0.062	21.139	0.175	15.2	15.8	16.9	17.6	18.8	21.1	23.8	25.3	26.4	28.1	29.3
9.0–9.9	0.129	24.532	0.163	18.0	18.7	19.9	20.7	22.0	24.5	27.4	29.0	30.1	31.9	33.1
10.0–10.9	0.220	28.173	0.151	21.0	21.8	23.1	24.0	25.4	28.2	31.2	32.9	34.1	35.9	37.1
11.0–11.9	0.323	31.981	0.140	24.3	25.2	26.6	27.6	29.0	32.0	35.1	36.9	38.1	40.0	41.2
12.0–12.9	0.412	35.892	0.130	27.7	28.7	30.2	31.2	32.8	35.9	39.1	40.9	42.2	44.1	45.3
13.0–13.9	0.439	39.827	0.120	31.4	32.4	34.0	35.1	36.7	39.8	43.1	44.9	46.2	48.1	49.4
14.0–14.9	0.355	43.580	0.110	35.2	36.2	37.7	38.8	40.4	43.6	46.9	48.7	50.0	51.9	53.2
15.0–15.9	0.169	46.820	0.100	38.6	39.6	41.1	42.2	43.7	46.8	50.1	51.9	53.2	55.1	56.4
16.0–16.9	−0.080	49.338	0.092	41.6	42.5	43.9	44.9	46.4	49.3	52.5	54.3	55.5	57.4	58.7
17.0–17.9	−0.352	51.284	0.084	44.0	44.8	46.2	47.1	48.5	51.3	54.3	56.0	57.2	59.1	60.3
18.0–18.9	−0.627	52.994	0.076	46.2	47.0	48.2	49.1	50.4	53.0	55.8	57.5	58.6	60.4	61.6
**LEAN MASS FOR EQUATION 2**														
5.0–5.9	−0.275	11.790	0.220	8.0	8.3	9.0	9.4	10.2	11.8	13.7	14.9	15.8	17.3	18.3
6.0–6.9	−0.139	15.053	0.207	10.3	10.8	11.6	12.2	13.1	15.1	17.3	18.7	19.7	21.3	22.4
7.0–7.9	0.028	18.168	0.193	12.6	13.2	14.2	14.9	15.9	18.2	20.7	22.2	23.3	24.9	26.1
8.0–8.9	0.206	21.212	0.180	14.9	15.6	16.7	17.5	18.8	21.2	23.9	25.5	26.6	28.3	29.4
9.0–9.9	0.351	24.405	0.168	17.5	18.3	19.5	20.4	21.7	24.4	27.3	28.9	30.0	31.7	32.9
10.0–10.9	0.433	27.861	0.156	20.4	21.2	22.6	23.6	25.0	27.9	30.9	32.6	33.7	35.5	36.7
11.0–11.9	0.470	31.533	0.144	23.6	24.5	26.0	27.0	28.5	31.5	34.7	36.4	37.6	39.5	40.7
12.0–12.9	0.467	35.381	0.133	27.1	28.1	29.6	30.7	32.3	35.4	38.6	40.4	41.7	43.6	44.8
13.0–13.9	0.403	39.364	0.122	31.0	31.9	33.5	34.6	36.2	39.4	42.7	44.5	45.8	47.7	49.0
14.0–14.9	0.257	43.251	0.111	34.9	35.9	37.4	38.5	40.1	43.3	46.6	48.4	49.7	51.7	53.0
15.0–15.9	0.030	46.687	0.100	38.6	39.6	41.1	42.1	43.6	46.7	49.9	51.8	53.1	55.0	56.3
16.0–16.9	−0.256	49.428	0.090	41.8	42.7	44.1	45.1	46.5	49.4	52.6	54.3	55.6	57.5	58.8
17.0–17.9	−0.570	51.570	0.081	44.6	45.3	46.6	47.5	48.9	51.6	54.5	56.2	57.4	59.2	60.5
18.0–18.9	−0.887	53.438	0.072	47.0	47.7	48.9	49.7	51.0	53.4	56.2	57.7	58.8	60.6	61.7

*LMS, Least-Mean-Square algorithm; L, Box-Cox coefficient; M, median; S, coefficient of variation; DXA, double energy X-ray absorptiometry*.

**Table 6:** LMS values and percentile distribution to assess muscle mass with DXA and anthropometric equations in females.

**Females**														
**Age**	**L**	**M**	**S**	**P3**	**P5**	**P10**	**P15**	**P25**	**P50**	**P75**	**P85**	**P90**	**P95**	**P97**
**LEAN MASS BY DXA**														
5.0–5.9	−2.013	14.628	0.202	11.0	11.3	11.9	12.3	13.0	14.6	17.1	19.2	21.1	25.3	30.0
6.0–6.9	−1.550	16.336	0.197	12.2	12.6	13.2	13.7	14.5	16.3	19.0	20.9	22.5	25.6	28.4
7.0–7.9	−1.090	18.122	0.193	13.4	13.8	14.6	15.1	16.0	18.1	20.8	22.7	24.2	26.7	28.7
8.0–8.9	−0.631	20.198	0.188	14.7	15.2	16.1	16.8	17.9	20.2	23.0	24.9	26.2	28.5	30.1
9.0–9.9	−0.177	22.842	0.183	16.3	17.0	18.1	19.0	20.2	22.8	25.9	27.7	29.0	31.1	32.6
10.0–10.9	0.241	26.076	0.178	18.4	19.2	20.6	21.6	23.1	26.1	29.4	31.2	32.6	34.6	36.0
11.0–11.9	0.581	29.565	0.174	20.6	21.6	23.3	24.4	26.2	29.6	33.1	35.1	36.4	38.5	39.9
12.0–12.9	0.801	32.691	0.169	22.7	23.9	25.8	27.1	29.0	32.7	36.5	38.5	39.9	42.0	43.4
13.0–13.9	0.880	34.867	0.164	24.3	25.6	27.6	29.0	31.0	34.9	38.8	40.9	42.3	44.4	45.8
14.0–14.9	0.843	36.120	0.159	25.6	26.9	28.9	30.2	32.3	36.1	40.0	42.2	43.6	45.8	47.2
15.0–15.9	0.704	36.679	0.155	26.5	27.7	29.6	30.9	32.9	36.7	40.6	42.7	44.2	46.3	47.8
16.0–16.9	0.481	36.803	0.150	27.2	28.3	30.1	31.3	33.2	36.8	40.6	42.7	44.2	46.4	47.9
17.0–17.9	0.206	36.615	0.145	27.7	28.7	30.3	31.4	33.2	36.6	40.3	42.5	43.9	46.2	47.7
18.0–18.9	−0.085	36.322	0.140	28.0	28.9	30.4	31.4	33.1	36.3	39.9	42.0	43.5	45.8	47.4
**LEAN MASS FOR EQUATION 1**														
5.0-5.9	−0.282	13.160	0.164	9.8	10.1	10.7	11.1	11.8	13.2	14.7	15.7	16.4	17.4	18.2
6.0-6.9	−0.089	15.645	0.160	11.6	12.1	12.8	13.3	14.0	15.6	17.4	18.5	19.2	20.4	21.2
7.0-7.9	0.101	18.103	0.156	13.4	14.0	14.8	15.4	16.3	18.1	20.1	21.3	22.1	23.3	24.2
8.0-8.9	0.272	20.703	0.152	15.4	16.0	16.9	17.6	18.7	20.7	22.9	24.2	25.0	26.4	27.3
9.0-9.9	0.405	23.567	0.148	17.5	18.2	19.3	20.1	21.3	23.6	26.0	27.3	28.3	29.7	30.7
10.0-10.9	0.509	26.584	0.144	19.9	20.7	21.9	22.8	24.1	26.6	29.2	30.7	31.7	33.2	34.2
11.0-11.9	0.582	29.497	0.140	22.2	23.1	24.4	25.4	26.8	29.5	32.3	33.9	35.0	36.6	37.7
12.0-12.9	0.613	31.964	0.135	24.2	25.2	26.6	27.6	29.1	32.0	34.9	36.6	37.7	39.4	40.5
13.0-13.9	0.577	33.727	0.131	25.8	26.8	28.3	29.3	30.8	33.7	36.8	38.4	39.6	41.3	42.5
14.0-14.9	0.440	34.881	0.127	27.1	28.0	29.5	30.5	32.0	34.9	37.9	39.6	40.8	42.6	43.8
15.0-15.9	0.169	35.537	0.123	28.1	28.9	30.3	31.2	32.7	35.5	38.6	40.3	41.5	43.4	44.6
16.0-16.9	−0.209	35.854	0.119	28.8	29.6	30.9	31.7	33.1	35.9	38.9	40.6	41.9	43.8	45.1
17.0-17.9	−0.651	35.906	0.115	29.3	30.0	31.2	32.0	33.3	35.9	38.9	40.6	41.9	43.9	45.3
18.0-18.9	−1.112	35.824	0.111	29.7	30.4	31.4	32.2	33.3	35.8	38.7	40.5	41.8	43.9	45.4
**LEAN MASS FOR EQUATION 2**														
5.0–5.9	−1.063	13.744	0.175	10.4	10.7	11.2	11.6	12.3	13.7	15.6	16.8	17.8	19.4	20.6
6.0–6.9	−0.728	16.051	0.171	12.0	12.4	13.1	13.6	14.4	16.1	18.1	19.4	20.4	22.0	23.2
7.0–7.9	−0.419	18.411	0.167	13.7	14.2	15.0	15.6	16.5	18.4	20.7	22.0	23.0	24.7	25.8
8.0–0.9	−0.155	21.011	0.163	15.6	16.2	17.1	17.8	18.8	21.0	23.5	24.9	26.0	27.6	28.8
9.0–9.9	0.054	23.953	0.159	17.7	18.4	19.5	20.3	21.5	24.0	26.7	28.2	29.3	31.1	32.2
10.0–10.9	0.216	27.140	0.155	20.1	20.9	22.2	23.0	24.4	27.1	30.1	31.8	33.0	34.8	36.0
11.0–11.9	0.334	30.310	0.151	22.5	23.4	24.8	25.8	27.3	30.3	33.5	35.3	36.6	38.5	39.8
12.0–12.9	0.399	33.096	0.147	24.7	25.7	27.2	28.3	29.9	33.1	36.5	38.4	39.7	41.7	43.0
13.0–13.9	0.391	35.153	0.143	26.5	27.5	29.1	30.2	31.9	35.2	38.6	40.6	41.9	44.0	45.4
14.0–14.9	0.281	36.535	0.139	27.9	28.9	30.4	31.6	33.2	36.5	40.1	42.1	43.5	45.6	47.0
15.0–15.9	0.040	37.366	0.35	29.0	29.9	31.4	32.5	34.1	37.4	40.9	42.9	44.4	46.6	48.1
16.0–16.9	−0.302	37.847	0.31	29.9	30.7	32.1	33.1	34.7	37.8	41.4	43.5	44.9	47.2	48.8
17.0–17.9	−0.704	38.077	0.126	30.6	31.4	32.7	33.6	35.1	38.1	41.6	43.7	45.2	47.7	49.4
18.0–18.9	−1.125	38.191	0.122	31.1	31.9	33.1	33.9	35.3	38.2	41.6	43.8	45.4	48.0	49.8

*LMS, Least-Mean-Square algorithm; L, Box-Cox coefficient; M, median; S, coefficient of variation; DXA, double energy X-ray absorptiometry*.

## Discussion

The results for the first objective showed that chronological age and the anthropometric variables, such as weight, height, and arm circumference, correlated significantly with LM. These high correlations gave rise to the creation of four models for predicting LM in children and adolescents of both sexes (two for males and two for females).

The LM models predicted 83 to 88%. The SEE was <5%. Furthermore, the VIF oscillated between 3.0 and 8.2. These reflected values less than those established as the maximum limit (20). This demonstrated no presence of multi-collinearity as predictors.

These results are consistent with other studies that have indicated that anthropometric variables continue to be excellent predictors of body components. This is especially the case when the Electric Bio-impedance (21, 22) and DXA methods (23–25) are used as criteria.

Furthermore, no significant differences occurred between the criterion (DXA) with the four equations generated for both sexes. To confirm this pattern, the desirability reproducibility index (DRI) was used. This allowed for evaluating the agreement between the two readings in terms of precision and accuracy. The results indicated an excellent level of agreement (CCC = 0.90–0.93) according to Lin's index (18). In addition, the precision and accuracy described the DRI were similar to those of other studies that have developed regression equations for body composition based on anthropometric variables (11, 22).

The information obtained from this research is relevant for reproducing similar results in other contexts. The models here guarantee the robustness of the equations created (26).

With regard to the second objective of this study, reference percentiles were developed to evaluate LM by age and sex. In general, males presented greater LM than females, especially during adolescence. In fact, maturation (APHV) occurred in males at 14.09 ± 0.8 APHV and for females at 11.07 ± 0.7APHV. This indicated the early onset of puberty in the females compared to the males. Considerable variation occurred in muscle mass between the sexes and individuals. This may be due to the increase in the growth hormone and androgen concentrations during adolescence that starts during the growth spurt (27).

The percentiles proposed may be useful for assessing healthy muscle development in children and adolescents. Moreover, they may contribute to monitoring and identifying growth and loss of muscle mass, particularly in children with metabolic illnesses (14).

In general, research has shown that elevated LM values may increase sensitivity to insulin (28) and improvement in bone health (29). On the other hand, low LM values relate to metabolic risk factors and resistance to insulin (30–32), and including the risk of osteoporosis (11).

In this sense, in this study, the researchers proposed percentiles and Z scores to classify LM not only based on regression equations, but also based on real DXA values. This simplifies the use, not only in laboratory contexts (based on the use of DXA in clinical settings), but also in the field (in epidemiological contexts and school environments). Furthermore, these may be used as a base line for analyzing pediatric patients, and they may be useful for future research (33).

The cut-off points adopted for this study were ≥p85 as excellent; p10 to p85 as good; p5 to <p10 as insufficient, and <p5 as serious. Although no consensus exists about a standard cut-off point for classifying sarcopenia, some studies have shown various cut-off points by highlighting average values ±1SD (34) and ±2SD (35). These discrepancies are possibly due to ethnic variations observed on body composition in studies focusing on children and adolescents (33, 36). This is due to the fact that each region presents its own social, economic, and cultural characteristics that make up a specific country.

Regardless of the cut-off points adopted, the percentiles developed may be an alternative that takes into account these intercultural variations in LM. This alternative may help determine the risk and presence of sarcopenia as well as help develop interventions for modifying lifestyle factors (34).

Therefore, to maintain an optimal skeletal muscle mass during childhood may improve the maximum muscle mass. Thus, exercising the muscle-skeletal system is more sensitive during childhood than during adulthood and old age (37).

This research has some strengths. For instance, the fact that it is the first study carried out with a large Chilean student sample size. In addition, both the regression equations as well as the real DXA values are fundamental tools for identifying LM extremes in laboratory conditions and field contexts.

In fact, the values obtained with non-invasive methods, such as the case of the regression equations here based on the anthropometric variables, always showed slight discrepancies with regard to the reference method (DXA). However, in spite of this, we maintain that the equations proposed are still a highly reliable alternative to implement and use during the school years for students, especially in contexts where laboratories and sophisticated equipment do not exist to evaluate lean mass.

This research may also be reproduced in other contexts with similar characteristics. The significant correlations observed between the anthropometric variables and the absence of collinearity in each one of the models reflected the capacity to adjust to each body size according to age and sex These equations have the potential to help control and diagnose LM, independent of nutritional status. Calculations may be carried out using this link: http://www.reidebihu.net/masamusch.php.

In addition, this study presented some limitations. For example, the design for this study was cross-sectional. Thus, the results needed to be corroborated through a longitudinal study. Furthermore, it was not possible to carry out physical evaluations, specifically for the isometric strength and muscle resistance variables. As well, the physical activity parameters were not measured in this sample population. If these variables had been collected, they may have explained more effectively the results of this study. Future research needs to incorporate these variables to better explain with more specificity Chilean students.

In conclusion, the four proposed equations were acceptable in terms of precision and accuracy in estimating LM in children and adolescents. The percentiles generated with anthropometric equations and real DXA values are fundamental tools for monitoring and detecting muscular anomalies and risks during somatic growth and development of children and adolescents of both sexes in Chile.

## Data Availability

The datasets analyzed in this manuscript are not publicly available. Requests to access the datasets should be directed to rossaunicamp@gmail.com.

## Ethics Statement

This study was approved by the Ethics Committee of the Universidad Autónoma of Chile (protocol no. 238/2014) and the respective authorities of the educational centers. Written informed consent to participate in this study was provided by the participants' legal guardian/next of kin.

## Author Contributions

RG-C and MC participated in the conception and design of the study, data analysis, and interpretation. MC, MA, RG-C, and CA participated in the writing of the manuscript, critical review, and approval of the final version. CU-A, CL-R, and JP-C participated in the gathering/acquisition of data and approval of the final version of the text. JS-T and MR-P participated in the generation of the link for the calculation of the lean mass.

### Conflict of Interest Statement

The authors declare that the research was conducted in the absence of any commercial or financial relationships that could be construed as a potential conflict of interest.
